# Physical activity and risk of colon adenoma: a meta-analysis

**DOI:** 10.1038/sj.bjc.6606045

**Published:** 2011-02-08

**Authors:** K Y Wolin, Y Yan, G A Colditz

**Affiliations:** 1 Division of Public Health Sciences, Department of Surgery, Washington University School of Medicine in St Louis and Alvin J Siteman Cancer Center, 660 S Euclid Avenue, Box 8100, St Louis, MO 63110, USA

**Keywords:** physical activity, colon adenoma, colon polyp

## Abstract

**Background:**

Little evidence is available on the relation of physical activity with colon adenomas, a colon cancer precursor.

**Methods:**

We conducted a systematic literature review and meta-analysis of published studies (in English) through April 2010, examining physical activity or exercise and risk or prevalence of colon adenoma or polyp. Random effects models were used to estimate relative risks (RRs) and corresponding confidence intervals (CIs). A total of 20 studies were identified that examined the association and provided RRs and corresponding 95% CIs.

**Results:**

A significant inverse association between physical activity and colon adenomas was found with an overall RR of 0.84 (CI: 0.77–0.92). The association was similar in men (RR=0.81, CI: 0.67–0.98) and women (RR=0.87, CI: 0.74–1.02). The association appeared slightly stronger in large/advanced polyps (RR=0.70, CI: 0.56–0.88).

**Conclusion:**

This study confirms previous reports of a significant inverse association of physical activity and colon adenoma, and suggests that physical activity can have an important role in colon cancer prevention.

Convincing evidence exists for a causal, inverse association between physical activity and risk of colon cancer (International Agency for Research on Cancer WHO, 2002; World Cancer Research Fund/American Institute for Cancer Research, 2007b; Wolin *et al*, 2009). This association is plausibly supported by several biological mechanisms, including decreased inflammation, decreased insulin-like growth factor levels, reduced hyperinsulinemia and modulated immune function (Wolin *et al*, 2009). Fewer data are available with respect to physical activity and colon adenomas, the precursor lesion detected and removed during sigmoidoscopy or colonoscopy. Although numerous studies have examined this association, no comprehensive meta-analysis is available. A limited meta-analysis by the World Cancer Research Fund included three studies, and estimated physical activity was associated with a statistically significant 15% reduction in colon polyp risk (World Cancer Research Fund/American Institute for Cancer Research, 2007a). Estimation of the risk reduction associated with the physical activity is important for public health because lifestyle is associated with a decreased risk of colon cancer, even among those who have undergone colon cancer screening (Wei *et al*, 2009). Furthermore, evidence on smoking has suggested that risk may vary for colon polyps versus colon cancer (Botteri *et al*, 2008). We therefore conducted a meta-analysis to estimate the summary relative risk (RR) of colon polyps associated with physical activity.

## Materials and methods

We searched the literature using PubMed, CINAHL and Scopus for all studies on physical activity or exercise and colon polyps through April 2010. We employed the terms exercise and physical activity in combination with colon polyps using the terms colon polyp, colon adenoma, colorectal polyp, colorectal adenoma and adenomatous polyps. We also utilised a previous review of the data (Samad *et al*, 2005; Lee and Oguma, 2006; World Cancer Research Fund/American Institute for Cancer Research, 2007a) and manual searches of the reference lists of identified manuscripts. We included recurrent, incident and prevalent cases of colon polyps. We did not limit studies by type of physical activity or study sample demographics.

Our search yielded 89 potential articles. We excluded reviews, non-human studies, editorials/comments/letters to the editor, studies without colon polyps as an outcome, studies where physical activity was only included as a covariate (and thus no measure of association was presented), and where no metric for effect estimate precision (*P*-value, s.e., confidence interval (CI)) was provided. Combined with searches from the reference sections of manuscripts and previous reviews, this yielded 20 manuscripts. From each manuscript, we abstracted the sample size (including number of cases), gender, years of follow-up or type of control sample, case definition, physical activity domain, adenoma detection method, sample definition criteria and results. We also abstracted the variables that each study used in its most adjusted analysis. Data extraction was performed by a single investigator (KYW). Where studies included more than one type of physical activity without a summary measure, we included only leisure time physical activity, which is the major modifiable component of energy.

Previous meta-analyses have suggested that results for adenomatous polyps need to be presented separately from hyperplastic or malignant polyps. (Botteri *et al*, 2008) Although we did not restrict our analysis to studies where data was limited to adenomatous polyps, we did consider those results separately. Specifically, we excluded results for hyperplastic polyps where feasible. We also identified studies considered to be the ‘best approach’ using criteria similar to those used in a previous meta-analysis (Botteri *et al*, 2008), namely, studies that met all of the following: (1) limited the outcome to only adenomatous polyps; (2) all individuals received a full colonoscopy; and (3) the study population excluded anyone with inherited colorectal cancer syndromes, inflammatory bowel disease, a history of colon polyps or cancer, or a previous colon resection.

### Data analysis

Meta-analysis of random effects was used to allow for the heterogeneity of results across studies. (Mosteller and Colditz, 1996) Data were processed in SAS, and the analyses were performed using R-package ‘meta’ (SAS Institute Inc., Cary, NC, USA). A summary forest plot was generated in Stata (StataCorp LP, College Station, TX, USA). As most studies reported RRs or odds ratios (ORs) and their associated 95 percent CIs, we used these data as summary statistics for each study. First, we derived the s.e. of log (RR or OR) using the 95 percent CI, with the expression: (log (upper limit) – log (lower limit))/2^*^1.96. These s.es were used as weights for summary effect estimates in the meta-analysis. We visually examined publication bias using Funnel plots, and employed the rank correlation method to formally test for bias. (Begg and Mazumdar, 1994) Where studies reported results separately for men and women, we included both estimates when reporting the overall association. To evaluate the potential effects of limiting results to only adenomatous polyps, we conducted exploratory analysis in the subset of those studies. We also included results separately for large/advanced adenoma, if the data were presented as such in the original manuscript. We also conducted exploratory analyses limited to those studies defined as the ‘best approach’. To test sub-analysis differences (large *vs* all adenomas; best approach *vs* all studies), we partitioned ‘total heterogeneity’ into between-group and within-group heterogeneity, and used the ‘between-group’ heterogeneity index as the test statistic against *χ*^2^ distribution with 1 degree of freedom. (Cooper and Hedges, 1994).

## Results

We identified 20 studies of physical activity and colon adenomas (Table 1). (Kono *et al*, 1991, 1999; Little *et al*, 1993; Shinchi *et al*, 1994; Giovannucci *et al*, 1995, 1996; Sandler *et al*, 1995; Neugut *et al*, 1996; Enger *et al*, 1997; Lubin *et al*, 1997; Kahn *et al*, 1998; Boutron-Ruault *et al*, 2001; Colbert *et al*, 2002; Lieberman *et al*, 2003; Tiemersma *et al*, 2003; Hauret *et al*, 2004; Wallace *et al*, 2005; Larsen *et al*, 2006; Rosenberg *et al*, 2006; Hermann *et al*, 2009) Most collected physical activity information via questionnaire, with nine studies only collecting information on leisure activity. Studies often did not specify or query the reasons participants underwent colonoscopy or sigmoidoscopy, thus, cases included are both symptomatic and screening. Only two studies (Colbert *et al*, 2002; Wallace *et al*, 2005) included procedures for the study, both were in studies of polyp recurrence. All but two studies (Kahn *et al*, 1998; Rosenberg *et al*, 2006) reported results for adenomas separately from all polyps or limited results to adenomas. A total of 10 studies (Shinchi *et al*, 1994; Giovannucci *et al*, 1995, 1996; Lubin *et al*, 1997; Kono *et al*, 1999; Boutron-Ruault *et al*, 2001; Colbert *et al*, 2002; Lieberman *et al*, 2003; Wallace *et al*, 2005; Larsen *et al*, 2006) reported results separately for large or advanced adenomas.

**Figure 1:**
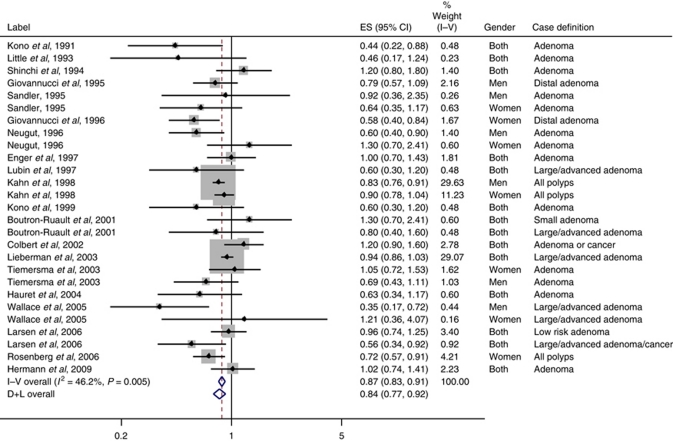
Meta-analysis of physical activity and colon adenoma. Study physical activity level comparisons are as follows: Kono *et al*, 1991: ⩾120 *vs* 0 min per week; Little *et al*, 1993: ⩾30 min *vs* none; Shinchi *et al*, 1994: daily *vs* none; Giovannucci *et al*, 1995: highest *vs* lowest quintile; Sandler, 1995: highest *vs* lowest quartile; Giovannucci *et al*, 1996: highest *vs* lowest quintile; Neugut *et al*, 1996: any *vs* none; Lubin *et al*, 1997: >5.5 h per day *vs* <4 h per day; Enger *et al*, 1997: highest *vs* lowest quartile; Kahn *et al*, 1998: high *vs* low; Kono *et al*, 1999: ⩾36 MET h per wk vs <4 MET h per wk; Boutron-Ruault *et al*, 2001: high *vs* low; Colbert *et al*, 2002: high *vs* low quartile; Lieberman *et al*, 2003: per 5 unit change in physical activity index; Tiemersma *et al*, 2003: not specified; Hauret *et al*, 2004: >40 MET h per wk *vs* <17.1 MET h per wk; Wallace *et al*, 2005: high *vs* low tertile; Larsen *et al*, 2006: high *vs* low quartile; Rosenberg *et al*, 2006: ⩾40 MET h per wk *vs* none; Hermann *et al*, 2009: active *vs* inactive. ES=effect size; MET=metabolic equivalent.

We found significant heterogeneity in the results (*P*<0.01) and thus, focus our report on the random effects analysis (Figure 1). Overall, there was a significant inverse association between physical activity and colon polyps (fixed effect RR=0.87, 95% CI: 0.83–0.91; random effects RR=0.84, 95% CI: 0.77–0.92) when comparing the most to least active individuals in each study. The summary RR was significant and similar in men (RR=0.81, 95% CI: 0.67–0.98) and women (RR=0.87, 95% CI: 0.74–1.02).

There was a tendency for the effect of physical activity to be restricted to large or advanced adenomas and not low grade ones. Similarly, physical activity was associated with large (>1 cm) (RR=0.63, 95% CI: 0.36–1.10), but not with small adenomas in a sample of US male health professionals (Giovannucci *et al*, 1995). In a cohort of US female nurses, a significant overall risk reduction (RR=0.58, 95% CI: 0.40–0.86) was reported, which was also stronger for larger than smaller adenomas (Giovannucci *et al*, 1996). Our meta-analysis found the effect was stronger, though not significantly so (*P*=0.16), for large or advanced (RR=0.70, 95% CI: 0.56–0.88) adenomas than for the overall effect. In analyses limited to the 18 studies where results for adenomatous polyps were separated from all polyps (i.e., hyperplastic, malignant polyps), the meta-analysis estimate for the association between physical activity and risk of polyps was largely unchanged (RR=0.83, 95% CI: 0.73–0.93). In analysis limited to the six studies (Kono *et al*, 1991; Sandler *et al*, 1995; Colbert *et al*, 2002; Lieberman *et al*, 2003; Tiemersma *et al*, 2003; Hauret *et al*, 2004) defined as the ‘best approach,’ the effect estimate was similar to that for all studies (RR=0.87, 95% CI: 0.73–1.05), though not statistically significant.

## Discussion

Previous, though limited, reviews have indicated physical activity is associated with a significant reduction in colon polyp risk. (World Cancer Research Fund/American Institute for Cancer Research, 2007a) Our comprehensive meta-analysis supports this conclusion, showing a significant 16% risk reduction when comparing the most to the least active. Risk reductions were similar for men and women, and held when limited to studies designated as the best approach. We found the association was notably stronger when analyses were limited to advanced or large polyps, with a risk reduction of 35%.

These results support the previously documented role of physical activity in colon cancer prevention (International Agency for Research on Cancer WHO, 2002; World Cancer Research Fund/American Institute for Cancer Research, 2007a and 2007b; Wolin *et al*, 2009). Earlier reports that failed to find an association between physical activity and colon polyps had suggested that physical activity may be more important in the adenoma to carcinoma sequence than in adenoma development (Colbert *et al*, 2002). Our meta-analysis, combined with the above-mentioned data demonstrating physical activity's role in colon cancer prevention, suggests that physical activity has a role across the carcinogenic process. Several mechanisms have been proposed for such effects, including enhanced immune function, decreased inflammation, reduced insulin levels and insulin resistance, and higher vitamin D levels (Wolin *et al*, 2009). Hyperinsulinemia has also been directly related to colon polyp risk (Wei *et al*, 2006).

This comprehensive meta-analysis provides support for an inverse association between physical activity and colon polyps, and also for the role of physical activity in colon cancer carcinogenesis. Physical activity may reduce the risk of colon polyps by 15% and may provide a substantially larger reduction in risk of large and advanced polyps.

## Figures and Tables

**Table 1:** Studies include in meta-analysis of physical activity and colon polyps

**Author and Year**	**Gender**	**Number of study subjects**	**Number of Cases**	**Relative Risk**	**Lower Confidence Interval**	**Upper Confidence Interval**	**Type of Physical Activity**	**Case definition**	**Non-case/ comparison definition**
Kono *et al*, 1991	Both	1148	80	0.44	0.22	0.87	Leisure	Adenoma	None
Little *et al*, 1993	Both	300	147	0.46	0.17	1.29	Leisure	Adenoma	FOBT negative
Shinchi *et al*, 1994	Both	1712	228	1.2	0.8	2	Leisure	Adenoma	None
Giovannucci *et al*, 1995	Men	12 879	455	0.79	0.57	1.09	Leisure	Distal Adenoma	No polyp
Sandler, 1995	Men	234	86	0.92	0.36	2.31	Leisure	Adenoma	Hyperplastic/none
Sandler, 1995	Women	350	114	0.64	0.35	1.19	Leisure	Adenoma	Hyperplastic/none
Giovannucci *et al*, 1996	Women	13 057	330	0.58	0.4	0.86	Leisure	Distal Adenoma	None
Neugut, 1996	Men	400	225	0.6	0.4	1	Total	Adenoma	None
Neugut, 1996	Women	411	283	1.3	0.7	2.3	Total	Adenoma	None
Enger *et al*, 1997	Both	920	460	1	0.7	1.5	Total	Adenoma	No polyp
Lubin *et al*, 1997	Both	392	196	0.6	0.3	0.9	Total	Large/advanced Adenoma	Hyperplastic/None
Kahn *et al*, 1998	Men	72 868	7504	0.83	0.76	0.91	Total	All polyps	None
Kahn *et al*, 1998	Women	81 356	5111	0.9	0.78	1.03	Total	All polyps	None
Kono *et al*, 1999	Both	415	189	0.6	0.3	1.3	Leisure	Adenoma	Normal
Boutron-Ruault *et al*, 2001	Both	581	154	1.3	0.7	2.5	Total	Small adenoma	None
Boutron-Ruault *et al*, 2001	Both	635	208	0.8	0.4	1.5	Total	Large/advanced adenoma	None
Colbert *et al*, 2002	Both	1839	733	1.2	0.9	1.6	Total	Adenoma or cancer	None
Lieberman *et al*, 2003	Both	2082	312	0.94	0.86	1.02	Total	Large/advanced adenoma	None
Tiemersma *et al*, 2003	Women	471	196	1.05	0.72	1.54	Not specified	Adenoma	None
Tiemersma *et al*, 2003	Men	398	237	0.69	0.43	1.1	Not specified	Adenoma	None
Hauret *et al*, 2004	Both	405	177	0.63	0.34	1.17	Total	Adenoma	Hyperplastic/None
Wallace *et al*, 2005	Men	787	539	0.35	0.17	0.72	Total	Large/advanced adenoma	None
Wallace *et al*, 2005	Women	787	205	1.21	0.36	4.03	Total	Large/advanced adenoma	None
Larsen *et al*, 2006	Both	3696	426	0.96	0.74	1.25	Total	Low risk adenoma	None
Larsen *et al*, 2006	Both	3376	106	0.56	0.34	0.92	Total	Large/advanced adenoma or cancer	None
Rosenberg *et al*, 2006	Women	45 400	1390	0.72	0.57	0.91	Leisure	All polyps	None
Hermann *et al*, 2009	Both	4510	527	1.02	0.74	1.42	Total	Adenoma	None

Abbreviation: FOBT=Fecal occult blood test.
